# Unveiling the Pharmacological Significance of Marine *Streptomyces violaceusniger* KS20: Isolation, Characterization, and Assessment of Its Biomedical Applications

**DOI:** 10.3390/metabo13091022

**Published:** 2023-09-19

**Authors:** Bidhayak Chakraborty, Kariyellappa Nagaraja Shashiraj, Raju Suresh Kumar, Meghashyama Prabhakara Bhat, Dhanyakumara Shivapoojar Basavarajappa, Abdulrahman I. Almansour, Karthikeyan Perumal, Sreenivasa Nayaka

**Affiliations:** 1 P.G. Department of Studies in Botany, Karnatak University, Dharwad 580003, India; bidhayak.20@kud.ac.in (B.C.); shashiraj@kud.ac.in (K.N.S.); meghashyama@kud.ac.in (M.P.B.); dhanyakumara@kud.ac.in (D.S.B.); 2 Department of Chemistry, College of Science, King Saud University, P.O. Box. 2455, Riyadh 11451, Saudi Arabia; sraju@ksu.edu.sa (R.S.K.); almansor@ksu.edu.sa (A.I.A.); 3 Department of Chemistry and Biochemistry, The Ohio State University, 151 W. Woodruff Ave, Columbus, OH 43210, USA; perumal.11@osu.edu

**Keywords:** *Streptomyces violaceusniger*, GC-MS analysis, antimycobacterial efficacy, antiproliferative activity, A549 cell line, PC-3 cell line

## Abstract

Marine actinomycetes represent a highly favorable source of bioactive compounds and have been the mainstay of much research in recent years. Recent reports have shown that marine *Streptomyces* sp. can produce compounds with diverse and potent biological activities. Therefore, the key objective of the study was to isolate and screen a potential actinomycete from marine ecosystems of Devbagh and Tilmati beaches, Karwar. *Streptomyces* sp. KS20 was characterized and the ethyl acetate extract (EtOAc-Ex) was screened for biomedical applications. *Streptomyces* sp. KS20 produced grayish-white aerial and pale-yellow substrate mycelia and revealed an ancestral relationship with *Streptomyces violaceusniger*. Optimum growth of the organism was recorded at 30 °C and pH 7.0. The metabolite profiling of EtOAc-Ex expressed the existence of several bioactive metabolites, whereas the functional groups were indicated by Fourier transform infrared (FTIR) spectroscopy. A considerable antioxidant activity was shown for EtOAc-Ex with IC_50_ of 92.56 μg/mL. In addition to this, *Streptomyces* sp. KS20 exhibited significant antimicrobial properties, particularly against *Escherichia coli*, where a zone of inhibition measuring 36 ± 0.83 mm and a minimum inhibitory concentration (MIC) of 3.12 µg/mL were observed. The EtOAc-Ex even revealed significant antimycobacterial potency with IC_50_ of 6.25 μg/mL. Finally, the antiproliferative potentiality of EtOAc-Ex against A549 and PC-3 cell lines revealed a constant decline in cell viability while raising the concentration of EtOAc-Ex from 12.5 to 200 μg/mL. The IC_50_ values were determined as 94.73 μg/mL and 121.12 μg/mL for A549 and PC-3 cell lines, respectively. Overall, the exploration of secondary metabolites from marine *Streptomyces* sp. KS20 represents an exciting area of further research with the potential to discover novel bioactive compounds that could be developed into therapeutics for various medical applications.

## 1. Introduction

Actinomycetes are the most vital assemblage of microorganisms and are believed to be the most biotechnologically and economically valuable prokaryotes [1]. From 1914 to 1939, an American inventor, microbiologist, and biochemist named Selman Waksman screened soil fungi, bacteria, and actinomycetes (*Actinomyces alboflavus*, *Actinomyces aureus*, *Actinomyces bobili*, *Actinomyces bovis*, *Actinomyces citreus*, *Actinomyces fradii*, *Actinomyces lavendulae*, etc.) to find a useful antibiotic for tuberculosis (TB) [2,3].

The vast majority of the Earth’s surface is occupied by the oceans and they are considered an inadequately inspected habitat in terms of microbial diversity, making them an ideal source for actinomycete isolation [4]. The relatively unexplored and underexplored habitats within marine ecosystems are widely regarded as promising reservoirs of rare actinomycetes, which possess significant potential for the production of novel and intriguing compounds. The marine actinomycetes exhibit great diversity in their habitats. They are found in marine and estuarine sediments, seawater, intertidal zones, and in symbiotic relationships with marine plants, invertebrates like echinoderms and sponges, vertebrates like puffer fish, etc. although the majority of strains have been isolated from marine sediments [5].

Actinomycetes are a class of microorganisms characterized by a complex life cycle, which possess the characteristics of both bacteria and fungi. Their name is derived from two Greek words, ‘aktis’ meaning ‘ray’ and ‘mukes’ meaning ‘fungi’. They are classified within the phylum Actinobacteria, constitute a substantial taxonomic group, and are presently acknowledged as part of the Bacteria domain [6]. The actinomycetes are Gram-positive bacteria and their DNA is composed of a significant proportion of cytosine and guanine (>55%). Great diversity in actinomycetes has been reported by many scientists. Some of the actinomycetes are rod or cocci shaped and some of them produce profusely branched mycelia to absorb nutrients and to produce spore-bearing structures [7].

Marine actinomycetes possess the capacity to generate a wide array of unique bioactive compounds with distinct functional and structural characters. This is due to extreme variations in availability of nutrients, high salinity, pressure, low temperature, etc. The competitive environment prevailing in the marine ecosystem has made the actinomycetes develop unique biochemical, physiological, and metabolic capabilities and also provides the potential to produce novel metabolites which are absent in terrestrial microorganisms [8].

Actinomycetes have huge economic importance in the production of enzymes, enzyme inhibitors, vitamins, novel pharmaceuticals, antitumor agents, antiparasitic agents, herbicides, pesticides, etc. [9]. Secondary metabolites produced by them exhibit an enormous number of compounds having biological activities. The order Actinomycetales is accountable for the synthesis of these biologically active metabolites with a remarkable record of over 10,000 antimicrobial compounds for medical uses [10]. Members of this order are the producers of several classes of antimicrobial substances, including β-lactams, aminoglycosides, macrolides, anthracyclines, glycopeptides, nucleosides, peptides, polyenes, polyketides, tetracyclines, actinomycin, and others [11,12].

Antimicrobial resistance (AMR) is an inherent process that arises when microorganisms come into contact with antimicrobial substances. Microbial pathogens persistently develop resistance to the actions of antimicrobial agents. A substantial decrease in antimicrobial research (between 1983 and 2007) has increased the severity of AMR and its consequences for worldwide healthcare [13]. Bacteria possessing innate resistance or those that have developed antimicrobial resistance traits are more likely to endure and reproduce successfully. The widespread increase in the utilization of easily accessible antibiotics has significantly contributed to AMR and further amplifies the probability of reappearance various diseases including TB [14,15].

Cancer encompasses a broad spectrum of diseases that have the potential to impact several areas of the body and accounted for nearly 10 million deaths in 2020. Worldwide, lung cancer (2.21 million cases) [16] is the second most common cancer while prostate cancer (1.41 million cases) in males ranked fourth in the total number of cases diagnosed in 2020 [17]. These incidents demand extensive research on the development of new anticancer compounds to decrease the occurrences and mortalities in the world.

Therefore, in the present investigation, an effort was made to screen and isolate a potential marine actinomycete from sediments and water samples from underexplored regions of Devbagh and Tilmati beaches, Karwar, Karnataka, India. The novelty of this work was that a marine actinomycete, *Streptomyces violaceusniger*, was isolated for the first time from a marine environment in India. This marine actinomycete was further characterized phenotypically and genotypically and investigated for its biological activities.

## 2. Materials and Methods

### 2.1. Pathogens and Materials Used in the Study

The pathogenic microorganisms were procured from the IMTECH, Chandigarh, India. Bacterial strains, including *Shigella flexneri* (MTCC 1457), *Pseudomonas aeruginosa* (MTCC 9027), *Escherichia coli* (MTCC 40), *Klebsiella pneumoniae* (MTCC 9238), *Bacillus subtilis* (MTCC 6633), *Staphylococcus aureus* (MTCC 6908), *Bacillus cereus* (MTCC 11778), and *Enterococcus faecalis* (MTCC 6845) were used. All the chemicals were bought from Himedia, laboratories, Mumbai, India.

### 2.2. Collection of Samples

Samples of seawater and sediments were gathered from different locations along the coastlines of Tilmati and Devbagh beaches in Karnataka, India. The samples were obtained randomly at a depth of 5 to 10 cm using aseptic techniques. They were then carefully stored in sterilized containers, labeled, and preserved at 4 °C until the actinomycete isolation process [18].

### 2.3. Isolation and Primary Screening

To isolate marine actinomycetes, the samples were diluted using sterilized physiological saline (0.9% NaCl) solution. Then, 100 µL suspensions from every dilution were evenly spread onto various media. These media were fortified with amphotericin-B and streptomycin (25 µg/mL) to avoid the growth of uninvited fungi and bacteria. At a temperature of 30 ± 2 °C, the plates were incubated for a period of 10 to 14 days, allowing actinomycete colonies to become visible [19].

The cross-streak method was employed to assess the antimicrobial capacity of the isolated actinomycetes. A single streak of the actinomycetes was grown on ISP-2 medium plates and incubated at 30 ± 2 °C for 7 days. Different types of microorganisms, such as Gram-negative and Gram-positive bacteria, along with yeasts, were streaked in a perpendicular manner to the actinomycete streak. The plates were then incubated for 7 days and at a temperature of 37 °C. Following the incubation period, the plates were assessed for antimicrobial activity. The actinomycete isolate showing potential activity was selected for further investigations [20].

### 2.4. Polyphasic Taxonomy of Streptomyces sp. KS20

#### 2.4.1. Morphological Characterizations

Morphological characterizations of *Streptomyces* sp. KS20 were conducted by documenting the color of aerial and substrate mycelia, staining nature, pigmentation, colony shapes, margins, and elevations. The mycelial and spore surface morphologies were examined using a scanning electron microscope (SEM) (JSM-IT500, In Touch Scope Scanning Electron Microscope, Tokyo, Japan) following the method of Divya and Nawani [21]. Briefly, *Streptomyces* sp. KS20 was fixed with 25% glutaraldehyde for 2 h, then washed thrice with PBS (pH 7.2 ± 0.2) and gradually dehydrated with increasing concentrations of acetone (30% to 100%), and finally dried in a critical point drier. For SEM analysis, the organism was subjected for 2 min to gold sputtering on conductive carbon tape and scanned using a SEM instrument at a resolution of 6000X.

#### 2.4.2. Molecular Characterization

*Streptomyces* sp. KS20 was taxonomically identified by gene sequencing of 16S rRNA. A HipurA *Streptomyces* DNA purification kit (#MB527) was utilized to extract and purify the genomic DNA following the producer’s instructions. The genomic DNA was then amplified with a reverse primer (1492R 5′-GGTTACCTTGTTACGACTT-3′) and forward primer (27F 5′-AGAGTTTGATCCTGGCTCAG-3′) using a PCR instrument (Applied Biosystems 2720 Thermal Cycler) [22]. Following amplification, the 16S rRNA gene was visualized and subsequently sequenced using a genetic analyzer. The acquired gene sequence was analyzed by comparing it to similar sequences in the NCBI database through the BLAST portal. Sequences with high similarity were used to construct a phylogenetic tree using MEGA7 software.

#### 2.4.3. Physiological Characterizations

Physiological characterizations of *Streptomyces* sp. KS20 involved observing its growth under various conditions: temperatures ranging from 20 to 45 °C, pH levels from 5.0 to 10.0, and sodium chloride concentrations (*w*/*v*) ranging from 1 to 7% [23].

#### 2.4.4. Biochemical Characterizations

The VITEK-2 Compact system (Biomerieux, Durham, NC, USA) with the BCL card was utilized to perform the biochemical analyses of *Streptomyces* sp. KS20. A suspension of the organism in 0.5 McFarland standards was applied to the microwell card, which contained specific test substrates [24]. The VITEK-2 BCL card revealed alkalization, acidification, growth inhibition, hydrolysis of enzymes, and assimilation of carbon sources for *Streptomyces* sp. KS20.

#### 2.4.5. Production and Extraction of Secondary Metabolites

*Streptomyces* sp. KS20 underwent submerged fermentation in starch casein (SC) broth (pH 7.0) for 20 days at 30 °C. After incubation, the biomass was filtered out of the broth, and the filtrate obtained was mixed with ethyl acetate (1:1, *v*/*v*) to facilitate the extraction of secondary metabolites. The separation of the ethyl acetate layer was carried out in a separating funnel after 24 h at room temperature and concentrated using a rotary evaporator at 40 °C under reduced pressure [25].

### 2.5. Characterizations of Ethyl Acetate Extract

#### 2.5.1. FTIR Spectroscopy

To detect the potential biological functional groups in the EtOAc-Ex of *Streptomyces* sp. KS20, we conducted FTIR analysis employing a Nicolet 6700 FTIR spectrophotometer (Thermo Fisher Scientific, Waltham, MA, USA). A small amount of EtOAc-Ex was ground with potassium bromide to prepare a thin disc, which was then scanned at a resolution of 4 cm^−1^ and in transmittance mode over the wavelength range of 4000 to 400 cm^−1^ [26].

#### 2.5.2. Gas Chromatography–Mass Spectrometry (GC-MS)

The volatile components in EtOAc-Ex of *Streptomyces* sp. KS20 were analyzed using a GC-MS system coupled with an electron ionization detector (Shimadzu GC-2010 Plus). The instrument consisted of an EC-5 column (0.25 μm film thickness, 15 m length, and 0.25 mm diameter). A 2 μL aliquot of EtOAc-Ex was introduced into a 2 mm injector with a split injection ratio of 10:1. The sample was transported using an inert helium gas with a consistent flow rate of 2 mL/min. Initial oven temperature was set to 35 °C for a duration of 2 min, after which it was ramped up to 450 °C at a rate of 20 °C/min. The analysis was performed for 43 min, covering a mass range of 65 *m*/*z* to 1000 *m*/*z*, in electron ionization mode. The mass spectra obtained were cross-checked with the National Institute of Standards and Technology (NIST) database to determine their identity [27].

### 2.6. Biological Activities

#### 2.6.1. Antioxidant Activity of *Streptomyces* sp. KS20 EtOAc-Ex

The potential of *Streptomyces* sp. KS20 EtOAc-Ex to scavenge free radicals was evaluated in vitro using 2,2-diphenyl-1-picrylhydrazyl (DPPH). Butylated hydroxytoluene (50 mg/mL) in methanol served as the standard, and a working solution of DPPH (0.024 g in 100 mL) was prepared. Several concentrations (25, 50, 75, 100, and 125 μg/mL) of BHT and EtOAc-Ex were separately pipetted, and DPPH solution (3 mL) was added to each. The combinations were then incubated for 30 min at room temperature in the dark. Following incubation, the measurement of absorbance at a wavelength of 517 nm was accomplished. The analysis was conducted in triplicate, and the findings were conveyed as the IC_50_ (µg/mL) [28]. The scavenging potential percentage was determined employing the following equation:

Radical scavenging activity = A_0_ − A_1_/A_0_ × 100

where A_0_ = absorbance of the control and A_1_ = absorbance of the sample.

#### 2.6.2. Antibacterial Activity of *Streptomyces* sp. KS20 EtOAc-Ex

The antibacterial efficacy of *Streptomyces* sp. KS20 was evaluated utilizing the agar well diffusion technique on nutrient agar (NA) (#MM012, Hi-media). EtOAc-Ex was dissolved in DMSO (10 mg/mL), the positive control used for bacteria was streptomycin (10 mg/mL). Pathogens including *S. aureus*, *B. cereus*, *B. subtilis*, *E. faecalis*, *E. coli*, *P. aeruginosa*, *S. flexneri*, and *K. pneumoniae* were cultured and adjusted to a 0.5 McFarland concentration (1.5 × 10^8^ CFU/mL). Subsequently, 100 µL of each bacterium was swabbed onto NA, then 6 mm wells were filled with 100 µL of EtOAc-Ex. A negative control was established using sterile distilled water, and the plates were subjected to incubation for 24 h at a temperature of 37 °C. The assay was performed in triplicate, and the zone of inhibition including the 6 mm well diameter for each pathogenic bacterium was calculated. The resultant zones of inhibition were then determined excluding the diameter (6 mm) of wells [29].

#### 2.6.3. Assessment of Minimum Inhibitory and Minimum Bactericidal Concentration

MIC was carried out according to CLSI microdilution guidelines. Briefly, nutrient broth (100 µL) was distributed in columns 1 to 12 and 100 µL of *Streptomyces* sp. KS20 EtOAc-Ex in DMSO (1 mg/mL) was two-fold serially diluted (100, 50, 25, 12.5, 6.25, 3.12, 1.6, 0.8, 0.4, 0.2 µg/mL) up to column 10. Fifty microliters of bacterial pathogens (0.5 McFarland concentrations, 1.5 × 10^8^ CFU/mL) was mixed in separate rows from columns 1 to 11. Columns 11 and 12 were considered as the bacterial growth control and sterility control, respectively. The plates were incubated at 37 °C for 24 h and, following incubation, 30 µL resazurin (0.015%) was added in each well. Incubation was carried out for 2 to 4 h for the examination of changes in color. For the determination of MBC, NA media were swabbed with pathogenic bacterial solutions from each well and incubated for 24 h at 37 °C. Plates with no bacterial colonies were determined as MBC [30].

#### 2.6.4. Antimycobacterial Activity

*Mycobacterium tuberculosis* strain H37 RV (ATCC 27294) was taken to measure antimycobacterial activity utilizing the microplate alamar blue assay (MABA). The peripheral wells of 96-well plates received 200 µL of sterile distilled water. One hundred microliters of Middlebrook 7H9 broth was poured into wells of column 1 to column 11 and 50 µL of the pathogen was mixed in each except column 11. In order to conduct the analysis, 5 common antimycobacterial medicines (isoniazid, ethambutol, pyrazinamide, rifampicin, and streptomycin) were used. Drugs and EtOAc-Ex were diluted serially from 100 to 0.2 µg/mL concentrations. The microplate was then incubated at 37 °C with 10% Tween 80 for 5 days. After incubation, 25 μL of alamar blue reagent (1:1) was added. Bacterial growth was indicated by a transition in color from blue to pink. The MIC was established as the concentration at which the occurrence of a color change was prevented [31].

#### 2.6.5. Antiproliferative Activity of *Streptomyces* sp. KS20 EtOAc-Ex

Human epithelial adenocarcinoma (A549), prostate adenocarcinoma (PC-3), and normal human embryonic kidney (HEK-293) cell lines were obtained from the National Centre for Cell Science (NCCS), Pune, India. Culturing of the cell lines was carried out in DMEM (#AL111, Himedia) supplemented with 10% FBS (#RM10432, Himedia) at 37 °C for 24 h in a 5% CO_2_ incubator. Once the cells reached full growth, approximately 20,000 cells/200 µL were transferred to separate 96-well microtiter plates. The 3-(4,5-dimethylthiazol-2-yl)-2,5-diphenyltetrazolium bromide (MTT) technique was employed for antiproliferative assay. The A549 cell line was employed, with the standard anticancer drug cisplatin (10 μM/mL) serving as a positive control, doxorubicin (10 µM/mL) for the PC-3 cell line, and camptothecin (10 µM/mL) for HEK-293 cell line. Cells with no treatment were designated as negative controls. Various concentrations of *Streptomyces* sp. KS20 EtOAc-Ex (12.5, 25, 50, 100, and 200 µg/mL) and anticancer drugs were introduced into the designated wells, and then the plates were incubated for 48 h at 37 °C in 5% atmospheric CO_2_. Following the incubation period, 50 µL of MTT reagent (5 mg/mL in PBS) was introduced to every well and incubated for 3 h. Subsequently, the produced formazan crystals were solubilized with DMSO (100 µL). Determination of absorbance was carried out at 570 nm and reference wavelength was set at 630 nm. The viability of cells was determined with the following formula: % cell viability = (OD of treated cells/OD of untreated cells) × 100. The IC_50_ was calculated using the following equation: Y = Mx + C [32].

## 3. Results

### 3.1. Isolation of Actinomycetes

In this study, we collected 36 marine samples from underexplored regions of Devbagh and Tilmati beaches, resulting in the isolation of 70 distinct actinomycetes.

### 3.2. Primary Screening of Antimicrobial Activity

Out of 70 marine actinomycetes, *Streptomyces* sp. KS20 expressed a good antibacterial activity against tested pathogens during the cross-streak method. In the case of *Streptomyces* sp. KS20, the pathogen *P. aeruginosa* did not show any inhibition, although other tested pathogens were susceptible.

### 3.3. Characterizations of Streptomyces sp. KS20

#### 3.3.1. Morphological Characterizations

**Figure 1:**
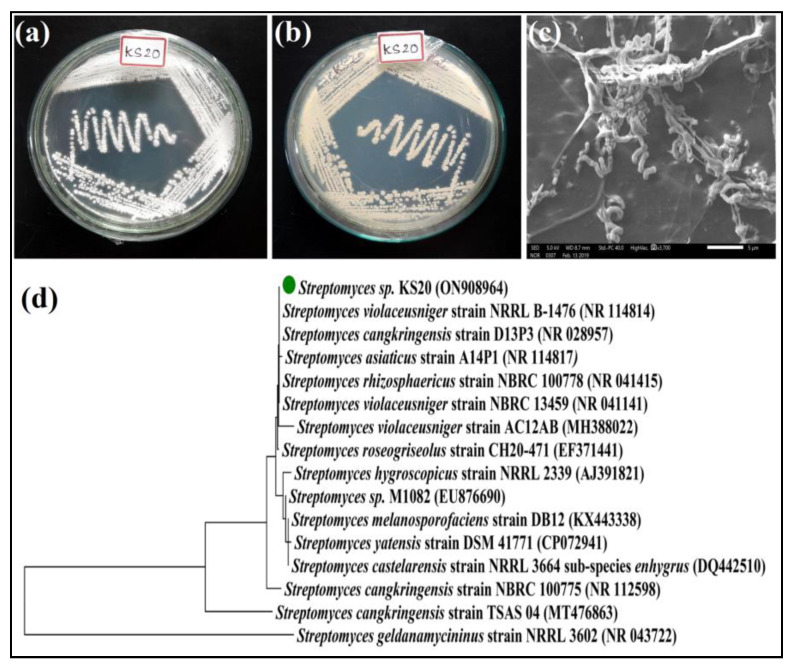
Characterizations of *Streptomyces* sp. KS20 on SA medium: (**a**) Grayish-white aerial mycelia; (**b**) pale-yellow substrate mycelia; (**c**) SEM image showing spiral spore chains and rugose spore surface; and (**d**) phylogenetic tree of *Streptomyces* sp. KS20 indicating the ancestral correlation with *Streptomyces violaceusniger* strain NRRL B-1476.

*Streptomyces* sp. KS20 was cultured on SA medium to carry out morphological characterization. This Gram-positive strain exhibited grayish-white aerial mycelia (Figure 1a) and pale-yellow substrate mycelia (Figure 1b). The colonies appeared dry, powdery, and circular in shape. Moreover, the organism displayed a pale-yellow pigmentation in the medium. The SEM analysis of the organism revealed spiral spore chains and the spores had rugose spore surfaces (Figure 1c). These spores were slightly curved and turned moist and black in color upon reaching maturity.

#### 3.3.2. Molecular Phylogeny of *Streptomyces* sp. KS20

In the case of *Streptomyces* sp. KS20, the 16S rRNA gene was 744 base pairs in length and a unique accession number, ON908964, was assigned to it in the NCBI database. BLAST analysis was performed for the gene and it was found to share 99.87% sequence similarity with *Streptomyces violaceusniger* strain NRRL B-1476 (NR114814). The evolutionary tree constructed with the neighbor-joining method involving 16 nucleotide sequences revealed the ancestral relationship between *Streptomyces* sp. KS20 and *Streptomyces violaceusniger* strain NRRL B-1476 (Figure 1d). Consequently, *Streptomyces* sp. KS20 was identified as *Streptomyces violaceusniger* strain KS20.

#### 3.3.3. Physiological Characterizations

*Streptomyces* sp. KS20 could grow best at 3 to 5% NaCl concentrations and other concentrations showed weak or no growth. Optimum pH for the growth of *Streptomyces* sp. KS20 was found at pH 7.0 and moderate to no growth was observed below and above pH 7.0. The temperature required for optimum growth was found to be 30 °C, and a weak growth was found at 25 °C and 35 °C. No growth was observed below 25 °C and above 35 °C (Table 1).

#### 3.3.4. Biochemical Characterizations

Detailed qualitative biochemical characterizations of *Streptomyces* sp. KS20 are listed in Table 2. The organism showed negative results for 26 tests and positive results for 20 tests. *Streptomyces* sp. KS20 exclusively utilized D-mannose as its carbon source. It exhibited several positive enzyme activities but was unable to grow in the presence of 6.5% NaCl. The organism displayed complete susceptibility to polymixin-B, oleandomycin, and kanamycin.

### 3.4. Fermentation and Extraction of Metabolites

Submerged fermentation was performed with *Streptomyces* sp. KS20 and, using equal volumes (1:1 *v*/*v*) of ethyl acetate, the secondary metabolites were extracted. The concentrated ethyl acetate yielded a yellow-colored oily extract.

### 3.5. Characterizations of EtOAc-Ex

#### 3.5.1. FTIR Spectroscopy

**Figure 2:**
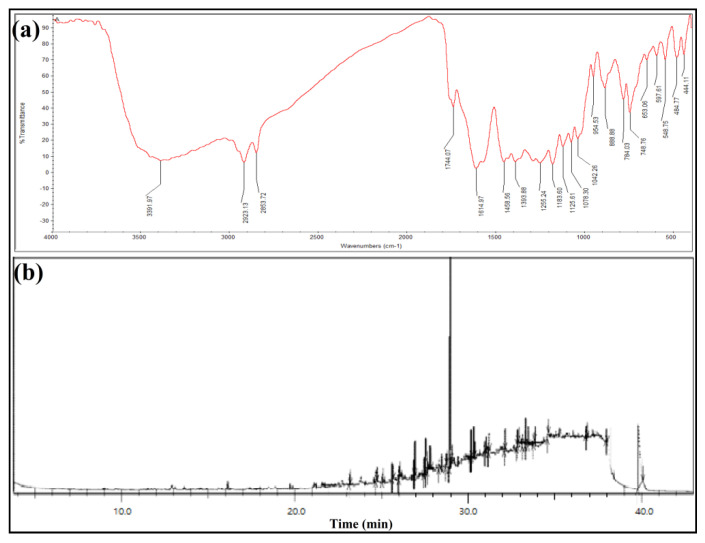
Characterizations of EtOAc-Ex of *Streptomyces* sp. KS20: (**a**) FTIR spectrum of EtOAc-Ex and (**b**) GC-MS chromatogram of EtOAc-Ex.

The FTIR spectrum of *Streptomyces* sp. KS20 EtOAc-Ex expressed twenty vibrational peaks corresponding to various functional groups (Figure 2a). The broad and strong peak at 3391.97 cm^−1^ could be assigned to O-H stretching of alcohol, and a sharp peak at 2923.13 cm^−1^ was indicative of C-H stretching of alkane functional groups. The vibrational peak at 2853.72 cm^−1^ appeared due to the C-H asymmetric/symmetric stretching of methylene. The medium and sharp peak at 1744.07 cm^−1^ was assigned to C=O stretching of esters, and the medium peak at 1614.97 cm^−1^ was assigned to C=C stretching of conjugated alkene. The absorption peak at 1458.56 cm^−1^ was ascribed to C=C-C ring stretching of aromatic functional groups and the weak peak at 1393.88 cm^−1^ was ascribed to O-H bending of carboxylic acids. A vibrational peak 1183.60 cm^−1^ was ascribed to C-O stretching of alcohols, the peak at 1125.61 cm^−1^ was credited to C-O stretching of aliphatic ether, and the peak at 1078.30 cm^−1^ appeared because of C-O stretching of primary alcohol. The absorption peak at 1042.26 cm^−1^ was indicative of P-O-C stretch of aliphatic phosphates, and the peak at 954.53 cm^−1^ coincided with trans-C-H out-of-plane bending. The absorption peak at 888.88 cm^−1^ was assigned to C=C bending of alkenes, and the peak at 784.03 cm^−1^ was indicative of C-H bending of alkenes. The peak at 653.06 cm^−1^ arose due to C-S stretching of thioethers.

#### 3.5.2. GC-MS Analysis

GC-MS analysis discovered the presence of 23 compounds in the *Streptomyces* sp. KS20 EtOAc-Ex (Figure 2b). A detailed list of compounds is present in Table 3, along with retention times, area %, height %, chemical formulas, and molecular weights. The GC-MS chromatogram displayed five major compounds, n-nonadecanol-1 (27.66%), L-(+)-ascorbic acid 2,6-dihexadecanoate (5.84%), di-sec-butyl phthalate (4.91%), 7,9-di-tert-butyl-1-oxaspiro[4.5]deca-6,9-diene-2,8-dione (3.35%), and octacosyl acetate (2.91%).

### 3.6. Biological Activities

#### 3.6.1. DPPH Radical-Scavenging Assay of *Streptomyces* sp. KS20 EtOAc-Ex

**Figure 3:**
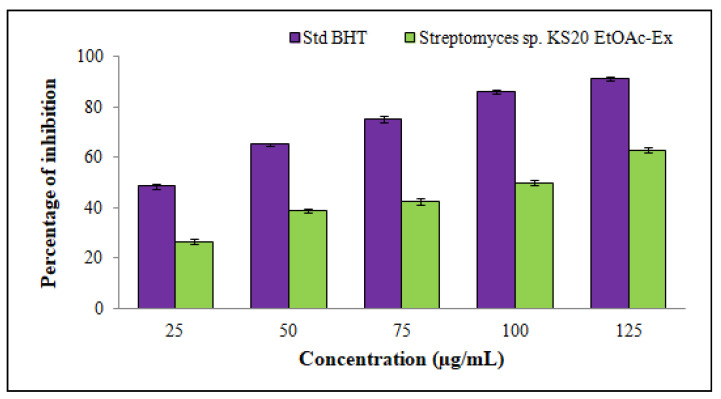
DPPH free radical-scavenging potential of *Streptomyces* sp. KS20 EtOAc-Ex.

A significant scavenging activity against DPPH free radicals was revealed by *Streptomyces* sp. KS20 EtOAc-Ex. The scavenging activity exhibited a positive correlation with the concentration of EtOAc-Ex, displaying a direct and dose-dependent relationship. This trend was observed as the concentration of EtOAc-Ex increased from 25 to 125 μg/mL (Figure 3). The percentage scavenging ratios were 26.39 ± 1.06%, 38.61 ± 0.81%, 42.53 ± 1.21%, 49.92 ± 1.19%, and 62.89 ± 1.26%. The IC_50_ of EtOAc-Ex was 92.56 μg/mL.

#### 3.6.2. Antibacterial Assay of *Streptomyces* sp. KS20 EtOAc-Ex

**Figure 4:**
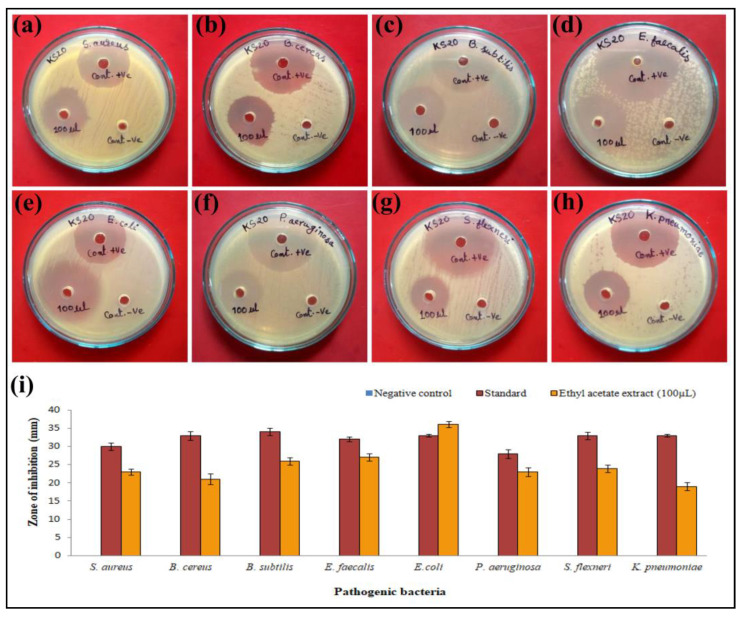
Antibacterial activity of EtOAc-Ex of *Streptomyces* sp. KS20: (**a**) *S. aureus*; (**b**) *B. cereus*; (**c**) *B.subtilis*; (**d**) *E. faecalis*; (**e**) *E. coli*; (**f**) *P. aeruginosa*; (**g**) *S. flexneri*; (**h**) *K. pneumoniae* and (**i**) bar graph indicating zone of inhibition (including 6 mm well diameter) against bacterial pathogens.

The EtOAc-Ex of *Streptomyces* sp. KS20 expressed significant activity against pathogenic bacteria (Figure 4a–h). The zones of inhibition (including 6 mm well diameter and excluding 6 mm well diameter) are represented in Table 4 for each tested bacterial pathogen. All tested pathogens expressed susceptibility to 100 µL EtOAc-Ex of *Streptomyces* sp. KS20. The highest inhibition activity (including 6 mm well diameter) was recorded against the bacterium *E. coli* (36 ± 0.83 mm) and lowest inhibition activity (including 6 mm well diameter) was observed against *K. pneumoniae*(19 ± 1.14 mm) (Figure 4i). The broth microdilution method revealed the MIC of EtOAc-Ex against all tested bacteria even at very low concentrations, which are represented in Table 5.

#### 3.6.3. Antimycobacterial Activity

**Figure 5:**
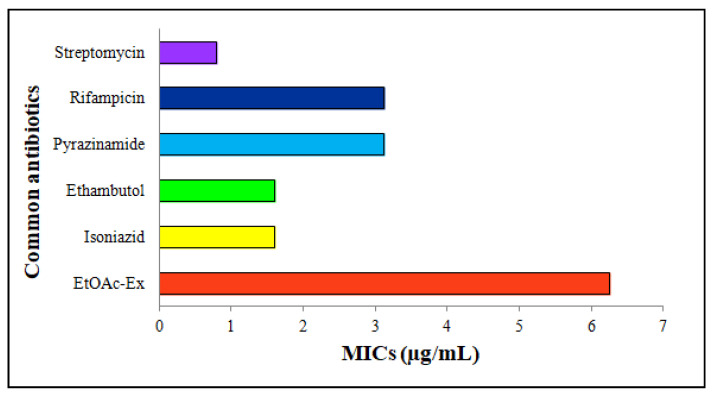
Antimycobacterial activity of *Streptomyces* sp. KS20 EtOAc-Ex by MABA method showing MIC of 6.25 μg/mL.

EtOAc-Ex expressed a moderate antimycobacterial activity. The common antibiotics and EtOAc-Ex were serially diluted from 100 to 0.2 μg/mL. Streptomycin, rifampicin, pyrazinamide, ethambutol, and isoniazid showed MICs of 0.8 μg/mL, 3.12 μg/mL, 3.12 μg/mL, 1.6 μg/mL, and 1.6 μg/mL, respectively. The MIC of EtOAc-Ex was 6.25 μg/mL (Figure 5).

#### 3.6.4. Antiproliferative Activity

**Figure 6:**
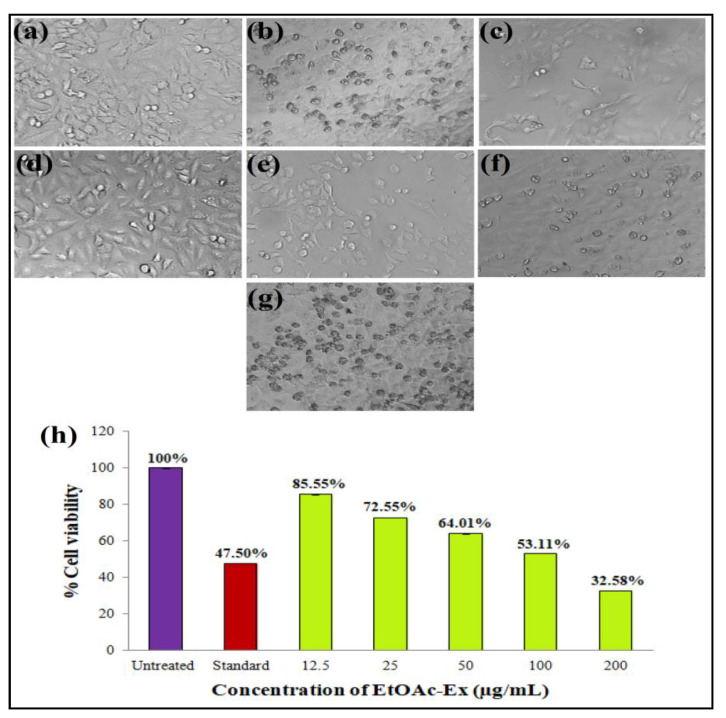
Antiproliferative activity of EtOAc-Ex of *Streptomyces* sp. KS20 against A549 cell line: (**a**) Negative control; (**b**) positive control; (**c**) 12.5 μg/mL; (**d**) 25 μg/mL; (**e**) 50 μg/mL; (**f**) 100 μg/mL; (**g**) 200 μg/mL; and (**h**) comparative % cell viability at different concentrations of EtOAc-Ex.

**Figure 7:**
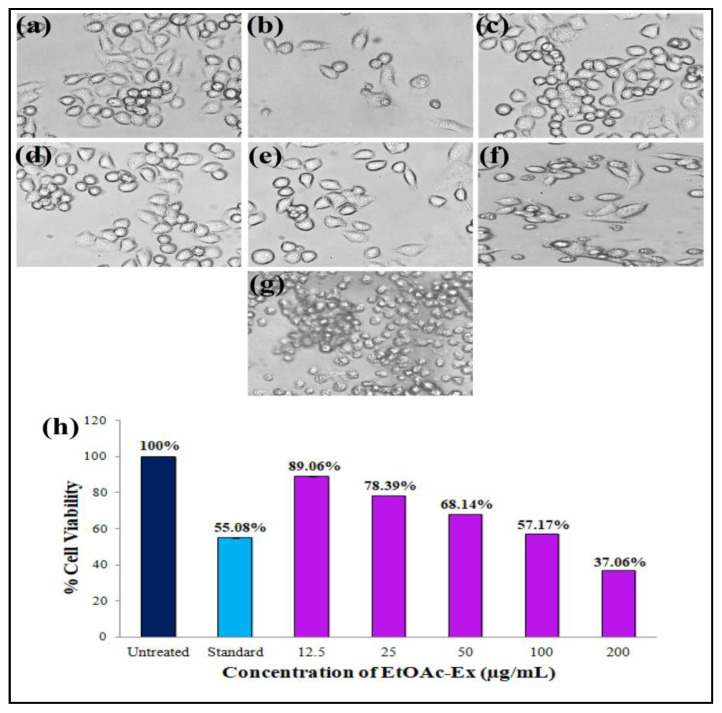
Antiproliferative activity of EtOAc-Ex of *Streptomyces* sp. KS20 against PC-3 cell line: (**a**) Negative control; (**b**) positive control; (**c**) 12.5 μg/mL; (**d**) 25 μg/mL; (**e**) 50 μg/mL; (**f**) 100 μg/mL; (**g**) 200 μg/mL; and (**h**) comparative % cell viability at different concentrations of EtOAc-Ex.

**Figure 8:**
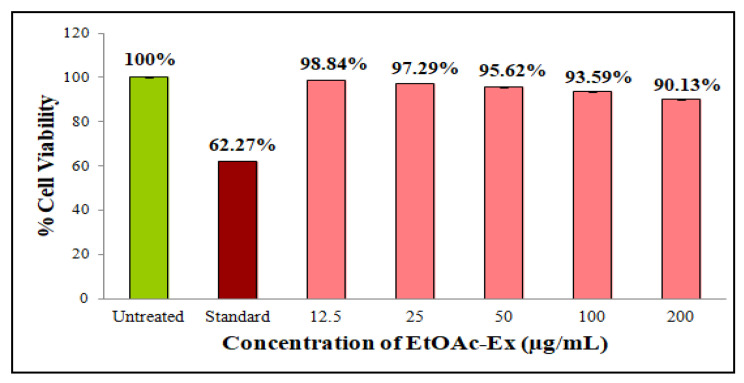
Comparative % cell viability of normal HEK-293 cell line at different concentrations of EtOAc-Ex.

In this work, the EtOAc-Ex of *Streptomyces* sp. KS20 was evaluated for antiproliferative potential against prostate cancer (PC-3) and lung cancer (A549) cell lines through an MTT assay. The A549 cells were subjected to treatment with various doses of EtOAc-Ex and the obtained result was compared with a standard chemotherapeutic drug cisplatin. Figure 6a,b represent the negative and positive controls, respectively. A gradual increase in cytotoxicity due to the increasing concentration of EtOAc-Ex is shown in Figure 6c–g. The cell viability was decreased to 97.78%, 88.02%, 73.12%, 61.64%, and 40.03% with increasing concentration of EtOAc-Ex of 12.5 to 200 μg/mL, respectively (Figure 6h). The IC_50_ of *Streptomyces* sp. KS20 EtOAc-Ex was 94.73 μg/mL for the A549 cancer cell line. In the case of the PC-3 cell line, the negative and positive controls are depicted in Figure 7a,b. A gradual decline in cell viability was recorded due to increasing concentration of EtOAc-Ex of *Streptomyces* sp. KS20 (Figure 7c–g). The cell viability was reduced to 96.75%, 82.64%, 65.19%, 50.44%, and 37.23% while treating with 12.5 to 200 μg/mL of EtOAc-Ex (Figure 7h). The IC_50_ was determined as 121.12 μg/mL for the PC-3 cancer cell line. The toxicity study with the normal HEK-293 cell line divulged a low toxicity of EtOAc-Ex after treatment for 24 h. The cell viability was determined as 98.84%, 97.29%, 95.62%, 93.59%, and 90.13% at 12.5, 25, 50, 100, and 200 μg/mL concentrations of EtOAc-Ex (Figure 8).

## 4. Discussion

Global health concerns in today’s world encompass the rise of uncontrolled diseases, multidrug-resistant human pathogens, resurgences of formerly subdued ailments, and inadequate therapeutic strategies to combat these emerging ailments [14]. Marine actinomycetes have surfaced as a promising reservoir of valuable substances, such as enzymes, antibiotics, and other bioactive metabolites that are industrially significant. However, research on actinomycetes from marine ecosystems remains limited and requires further exploration in the Indian subcontinent [45]. In this study, 70 distinct actinomycetes were collected from 36 marine samples of Devbagh and Tilmati beaches. The process of isolation of actinomycetes from unexplored environments has gained popularity as a means to fulfill the growing demand for novel antibiotics [24].

During the cross-streak method, *Streptomyces* sp. KS20 evidenced a potent antibacterial activity against the pathogenic microbes. In cross-streak method, *Streptomyces* sp. KS20 secreted antimicrobial compounds during its growth, which were distributed in the medium. It is suggested that the antimicrobial compounds are extracellular in nature and the diffusion of secreted metabolites in the medium occurs after the exponential growth period. This outcome demonstrated the synthesis of numerous antibacterial metabolites [46]. This finding agrees with the findings of Nayaka et al. [47], where *Streptomyces thermocarboxydus* isolated from the Kali River ecosystem could restrain the growth of pathogens during the screening process.

Morphologically, the colonies of *Streptomyces* sp. KS20 were powdery and circular in appearance. The substrate and aerial mycelia were pale yellow and grayish white in color, respectively. These characteristics are commonly employed for the initial identification of *Streptomyces* species [28]. It has been reported that various factors, such as carbon and nitrogen sources, temperature, pH, and trace elements of the culture medium, influence the color of mycelia and production of diffusible pigments [19,48]. The organism was subjected to SEM analysis, which revealed the presence of spiral spore chains and the spores exhibited rugose surfaces. A previous study reported a similar finding, where a soil actinomycete, *Streptomyces solisilvae*, produced spiral spore chains with rugose ornamentation [48].

Sequencing of the 16S rRNA gene is a highly effective tool for accurately identifying bacteria at the species level. It has been a mainstay of sequence-based bacterial analysis for decades for its capacity to distinguish between closely related bacterial species [19]. The 16S rRNA gene sequence of *Streptomyces* sp. KS20 disclosed 99.87% similarity with *Streptomyces violaceusniger* strain NRRL B-1476. As a result, *Streptomyces* sp. KS20 was determined as *Streptomyces violaceusniger* strain KS20. The 16S rRNA gene consists of highly conserved and hypervariable regions, forming part of the 30S small subunit of prokaryotic ribosomes. The hypervariable regions, due to slow rates of evolution, could retain species-specific signature sequences, which allow for bacterial identification, whereas the conserved regions serve as binding sites for universal primers [49]. This finding aligns with the research by Sreenivasa et al. [50], who identified *Streptomyces* sp. SN-3 as *Streptomyces gancidicus* through 16S rRNA gene sequencing.

During physiological characterization, *Streptomyces* sp. KS20 showed optimum growth at 30 °C and pH 7.0. Hence, it is reasonable to infer that the organism exhibited mesophilic and neutrophilic characteristics. These findings are similar to those of the research conducted by Nayer and Asmaa [29], who observed that *Streptomyces* sp. NMF6 thrived at pH levels from 4.0 to 10.0 and a temperature range of 20 to 45 °C and had a maximum tolerance of 4% NaCl concentration.

The biochemical analysis of *Streptomyces* sp. KS20 was carried out by VITEK2, which was necessary for accurate characterizations. A large number of enzymes are produced by bacteria, which allow their accurate identification through distinct enzymatic profiles. In addition, a variety of substrate utilization tests are available for identifying bacteria. The distinctive patterns of color changes in the substrates caused by bacteria can be used to identify them to the species level [51]. Out of 46 tests, *Streptomyces* sp. KS20 was positive in 20 tests and negative in 26 tests. It could assimilate the only carbon source, D-mannose, and displayed susceptibility to different antibiotics. VITEK2 is equipped with a transmittance optical system for effective interpretation of the test reactions while using various wavelengths in the visible spectrum [22]. This finding agreed with the report of Meghashyama et al. [52], where a *Streptomyces* sp. was biochemically characterized using the VITEK2 BCL card, which revealed 20 positive results for carbon source utilization, enzyme activities, and antibiotic susceptibility.

FTIR is a physicochemical method that provides a clear image of the metabolites by measuring the rotation and vibration of molecules in response to an infrared wavelength. The absorbed wavelength is the characteristic of a chemical bond, as it is reflected in the annotated spectrum. By the interpretation of the infrared spectrum, the functional groups and chemical bonds in a molecule can be determined [53]. The compounds present in EtOAc-Ex contained various functional groups like alcohol esters, aliphatics, carboxylic acids, aromatics, etc. A few other compounds were identified, having single bonds (alkanes) and double bonds (alkenes). The result obtained aligns with the findings reported by Chakraborty et al. [54], where FTIR spectroscopy analysis of ethyl acetate extract from *S. levis* indicated the existence of different types of functional groups, including carboxylic acids, alcohols, and esters.

The phylum Actinobacteria is widely acknowledged as a dynamic and prolific reservoir of diverse secondary bioactive compounds. A total of 23 compounds were reported through GC-MS analysis. The outcome is similar to the report of Janpen et al. [28], who used GC-MS to identify 24 compounds in ethyl acetate extract of the marine *Streptomyces achromogenes*.

Antioxidants are substances that prevent or delay cell damage by disarming unstable free radicals. In response to external and other factors, the body produces free radicals, which can make a person more susceptible to inflammation and a number of other illnesses [55]. During an antioxidant assay, BHT was used as a reference compound. It has a low molecular weight and a non-staining hindered-phenolic structure. Hindered phenols have a wide variety of applications, including inhibitors of free radical chain reactions. Its antioxidant properties are primarily attributed to its chemical structure and ability to scavenge free radicals and inhibit the propagation of oxidation reactions [56]. The EtOAc-Ex of *Streptomyces* sp. KS20 displayed noteworthy scavenging activity against DPPH free radicals through an effective dose-dependent relationship. The IC_50_ was determined as 92.56 μg/mL. DPPH is a stable free radical having a lone pair of electrons, which gives rise to a deep violet color. This color turned from violet to yellow when DPPH solution was mixed with *Streptomyces* sp. KS20 EtOAc-Ex containing antioxidant compounds. The acceptance of hydrogen atoms supplied by antioxidant compounds was the cause of this change in color. The change caused a decrease in absorbance values, which was quantitatively measured by recording the absorbance change [57]. This outcome was consistent with the conclusion of Dharaneedharan et al. [58], where ethyl acetate extract from *Streptomyces carpaticus*, a marine organism, expressed DPPH-scavenging activity (IC_50_ = 84.5 μg/mL).

A pronounced antibacterial potency was elicited by EtOAc-Ex of *Streptomyces* sp. KS20. The growth of all tested bacterial pathogens was suppressed even at low concentrations as evidenced by MIC. This could be due to the cumulative effect of a higher number of bioactive metabolites in the EtOAc-Ex. Resazurin sodium salt, a cell-permeable nontoxic dye, is widely used as a redox indicator in the MIC method. Resazurin salt changes its color based on metabolic activity of bacterial cells, which is important to determine the MIC. The appearance of a purple color indicated inhibition of microbial growth; whereas a pink color indicated actively growing cells, which reduce resazurin to resorufin [30]. *Streptomyces* spp. have enormous potential for the discovery of bioactive compounds, which can fight against resistant microorganisms. *Streptomyces* spp. possess immense possibilities for the discovery of bioactive substances that can combat antimicrobial-resistant pathogens [10]. Bioactive EtOAc-Ex is made up of a complex combination of ingredients, and their synergistic action can result in an enhanced antibacterial impact. They have a broad range of antimicrobial activity based on the location, structure, and number of substituent groups, the occurrence of OH group alkylations, glycosidic linkages, etc. However, differences in the qualities and quantities in the metabolites result in alterations to the effectiveness of antimicrobial activity against various microorganisms [59].

**Figure 9:**
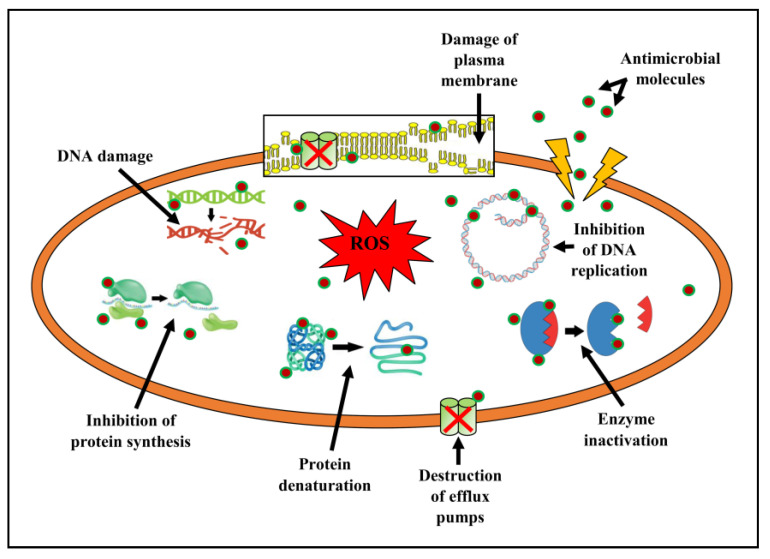
Antibacterial mechanism of actions of compounds present in EtOAc-Ex.

In pathogenic microbes, the antibacterial agents possess the capacity to interfere with the permeability of the membranes, cell wall biosynthesis, proteins, nucleic acid synthesis, etc. When there is a disruption in the cell membrane permeability, it leads to the alteration of a cellular ion gradient, and pathogens die as a result of cellular damage and exo- or endosmosis. Occasionally, the antimicrobials, after entering the plasma membrane, decimate bacterial cells. This is achieved by blocking the production of crucial substances, hindering protein synthesis and DNA replication, preventing the attachment of small subunits of rRNA, and repressing the efflux pumps (Figure 9) [60,61,62].

Mycobacterium tuberculosis is responsible for the infectious disease known as TB, which is a significant contributor to global mortality rates. According to a report from the WHO, 1.6 million people worldwide passed away from TB in 2021, making it the 13th most common cause of death globally. The primary target of the pathogen is the lungs although it eventually affects other organs [63]. Therefore, to deal with this problem, the antimycobacterial activity of *Streptomyces* sp. KS20 EtOAc-Ex was investigated by the MABA method. A moderate antimycobacterial activity was unveiled by EtOAc-Ex with MIC of 6.25 μg/mL. The antimycobacterial activity of the EtOAc-Ex was possibly mediated through mechanisms like inhibition of crucial enzymes, disruption of cell wall integrity, modulation of the immune responses, generation of reactive oxygen species (ROS), etc. This finding was in agreement with the study of Anuradha et al. [64], where ethyl acetate extract of *Streptomyces luridus* revealed a MIC of 1000 µg/mL against M. tuberculosis.

Cancer stands out as one of the most fatal diseases on a global scale. In an effort to find new anticancer drugs with fewer side effects, researchers are looking into natural sources, particularly marine resources. The genus *Streptomyces* has produced a number of anticancer drugs, including doxorubicin, dactinomycin, and bleomycin. Additionally, several studies have recorded the anticancer properties of crude extracts made from various marine *Streptomyces* spp. [65]. An antiproliferative assay was performed through the MTT assay. This assay is based on an enzyme called mitochondrial lactate dehydrogenase that reduces MTT. This enzyme exists within living cells and has the ability to transform yellow tetrazolium MTT into purple formazan crystals, which precipitate in the presence of healthy, unaffected cells [66]. The viability of A549 cells was reduced from 97.78 to 40.03% with various concentrations of EtOAc-Ex. For the A549 cancer cell line, the IC_50_ was calculated to be 94.73 μg/mL. During the treatment of PC-3 cells, the cell viability was gradually reduced from 96.75 to 37.23% with increasing concentration of EtOAc-Ex. The IC_50_ was 121.12 μg/mL for the PC-3 cancer cell line. This result indicated a significant antiproliferative potentiality of EtOAc-Ex against cancer cells. However, during the treatment of a normal eukaryotic cell line, the EtOAc-Ex exhibited a low toxicity and a slight decrease in cell viability was recorded from 98.84 to 90.13%. There are many studies that provide strong supporting evidence that *Streptomycetes* spp. are excellent sources for isolating anticancer-related compounds. Here, the EtOAc-Ex from *Streptomyces* sp. KS20 contains a wide variety of bioactive compounds that exhibited anticancer properties.

**Figure 10:**
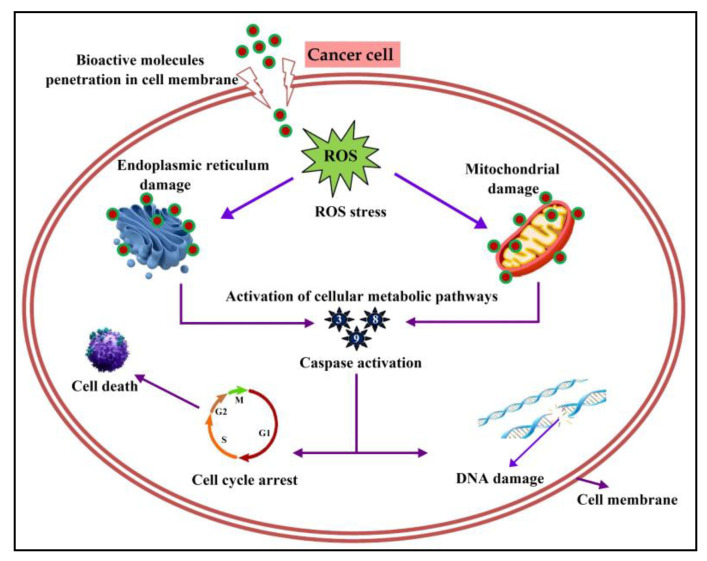
Mechanism of action of bioactive molecules from EtOAc-Ex against cancer cell.

The mechanisms by which cancer cells were killed can be diverse and multifaceted. The compounds present in EtOAc-Ex could activate specific signal pathways, such as the mitochondrial pathway or death receptor pathway, to trigger the apoptosis in tumor cells. Cancer cells often exhibit uncontrolled growth and division. Sometimes the compounds in the extract interfere with the cell cycle or inhibit the activity of proteins and various signaling pathways involved in cell proliferation. At times some bioactive compounds can directly damage the DNA of cancer cells, disrupting their ability to replicate and survive. Disruption of cell membrane integrity of cancer cells can also cause subsequent cell death. Bioactive metabolites can induce the generation of ROS within cancer cells. ROS can cause oxidative stress and damage to cellular components, ultimately leading to cell death. Specific signaling pathways which are involved in cancer cell growth and survival can also be damaged (Figure 10) [67,68,69]. A comparable outcome was reported by Balachandran et al. [70], where a *Streptomyces* sp. ethyl acetate extract revealed a dose-dependent cytotoxic activity with an IC_50_ of 600 µg/mL.

## 5. Conclusions

*Streptomyces* sp. KS20 was isolated from marine samples and selected based on antimicrobial potentiality against pathogens. *Streptomyces* sp. KS20 was described in terms of morphological, physiological, and biochemical methods. 16S rRNA gene sequencing of the organism disclosed 99.87% relatedness with *Streptomyces violaceusniger*. In addition to this, the secondary metabolites of *Streptomyces* sp. KS20 were extracted and subjected to molecular profiling and evaluated for a few biological activities. The EtOAc-Ex from *Streptomyces* sp. KS20 expressed a considerable antioxidant activity and a profound antimicrobial activity against pathogens. A promising antiproliferative activity was also revealed by the EtOAc-Ex of *Streptomyces* sp. KS20 against A549 and PC-3 cell lines. It is important to note that the EtOAc-Ex contains a number of compounds with potential biological properties. Additionally, it is crucial to conduct further research for purification, identification, and structure elucidation of the compounds having potential antioxidant, antimicrobial, and antiproliferative activities.

## Figures and Tables

**Table 1:** Physiological characterizations of *Streptomyces* sp. KS20.

Growth in Different NaCl Concentrations		Growth at Different pH		Growth at Different Temperatures	
Tests	Results	Tests	Results	Tests	Results
1%	−	pH 5.0	−	20 °C	−
2%	w	pH 6.0	+	25 °C	w
3%	++	pH 7.0	++	30 °C	++
4%	++	pH 8.0	w	35 °C	w
5%	++	pH 9.0	−	40 °C	−
6%	w	pH 10.0	−	45 °C	−
7%	−				

Key: − = no growth, w = weak growth, + = moderate growth, ++ = optimal growth.

**Table 2:** Biochemical characterizations of *Streptomyces* sp. KS20.

Tests	Amount per Well (mg)	Results	Tests	Amount per Well (mg)	Results
BETA-XYLOSIDASE	0.0324	−	D-MANNITOL	0.3	−
L-Lysine-ARYLAMIDASE	0.0228	+	D-MANNOSE	0.3	+
L-Aspartate ARYLAMIDASE	40.024	+	D-MELEZITOSE	0.3	−
Leucine ARYLAMIDASE	0.0234	+	N-ACETYL-D-GLUCOSAMINE	0.3	−
Phenylalanine ARYLAMIDASE	0.0264	+	PALATINOSE	0.3	−
L-Proline ARYLAMIDASE	0.0234	+	L-RHAMNOSE	0.3	−
BETA-GALACTOSIDASE	0.036	+	BETA-GLUCOSIDASE	0.036	+
L-Pyrrolidonyl-ARYLAMIDASE	0.018	−	BETA-MANNOSIDASE	0.036	−
ALPHA-GALACTOSIDASE	0.036	+	PHOSPHORYL CHOLINE	0.0366	+
Alanine ARYLAMIDASE	0.0222	+	PYRUVATE	0.15	−
Tyrosine ARYLAMIDASE	0.0282	+	ALPHA-GLUCOSIDASE	0.036	+
BETA-N-ACETYL-GLUCOSAMINIDASE	0.0408	+	D-TAGATOSE	0.3	−
Ala-Phe-Pro ARYLAMIDASE	0.0384	+	D-TREHALOSE	0.3	−
CYCLODEXTRIN	0.3	+	INULIN	0.12	−
D-GALACTOSE	0.3	−	D-GLUCOSE	0.3	−
GLYCOGEN	0.1875	−	D-RIBOSE	0.3	−
myo-INOSITOL	0.3	−	PUTRESCINE assimilation	0.201	−
METHYL-A-D-GLUCOPYRANOSIDE acidification	0.3	−	GROWTH IN 6.5% NaCl	1.95	−
ELLMAN	0.03	−	KANAMYCIN RESISTANCE	0.006	−
METHYL-D-XYLOSIDE	0.3	−	OLEANDOMYCIN RESISTANCE	0.003	−
ALPHA-MANNOSIDASE	0.036	+	ESCULIN hydrolysis	0.0225	+
MALTOTRIOSE	0.3	−	TETRAZOLIUM RED	0.0189	+
Glycine ARYLAMIDASE	0.012	+	POLYMIXIN_B RESISTANCE	0.00093	−

Key: − = negative, + = positive.

**Table 3:** GC-MS analysis of EtOAc-Ex of *Streptomyces* sp. KS20.

Compound Names	Retention Times	Area%	Chemical Formulas	Molecular Weights	Biological Activities	References
Trans-8-Methyl-1.beta.-acetyl-hydrindan	23.158	1.35	C_12_H_20_O	180.29	-	-
Tetradecanoic acid	24.666	1.26	C_14_H_28_O_2_	228.3	Nematicidal, antibacterial, and larvicidal	[33]
Pentadecanoic acid	25.618	1.70	C_15_H_30_O_2_	242.40	Anti-inflammatory, anticancer antifibrotic, red blood cell stabilizer	[34]
1,7-Dimethyl-4-(1-methylethyl)cyclodecane	26.009	1.30	C_15_H_30_	210.40	-	-
Heptadecanal	26.837	0.94	C_17_H_34_O	254.5	-	-
7,9-Di-tert-butyl-1-oxaspiro[4.5]deca-6,9-diene-2,8-dione	26.903	3.35	C_17_H_24_O_3_	276.4	Antioxidant, antimicrobial	[35]
L-(+)-Ascorbic acid 2,6-dihexadecanoate	27.555	5.84	C_38_H_68_O_8_	652.9	Antioxidant, antibacterial, antiviral, antiscorbutic, anti-inflammatory, anticancer, antimutagenic	[36,37]
Fumaric acid, isopropyl tetradecyl ester	28.309	1.34	C_21_H_38_O_4_	354.5	-	-
Hexadecane-1,2-diol	28.704	1.20	C_16_H_34_O_2_	258.44	-	-
n-Nonadecanol-1	28.947	27.66	C_19_H_40_O	284.5	Antimicrobial, anticancer	[38]
Nonadecyl pentafluoropropionate	30.142	2.49	C_22_H_39_F_5_O_2_	430.5	Antioxidant	[39]
Octacosyl acetate	30.310	2.91	C_30_H_60_O_2_	452.8	-	-
Heptacosyl acetate	30.949	0.81	C_29_H_58_O_2_	438.8	-	-
Triarachine	31.192	1.82	C_63_H_122_O_6_	975.63	Plays an important role in metabolism as energy source	[40]
1-Heptacosanol	32.089	1.64	C_27_H_56_O	396.73	Antimicrobial, antidiabetic, antioxidant, nematocidal	[41]
Methyl 5(Z),11(Z),14(Z)-Eicosatrienoate	32.785	0.54	C_21_H_36_O_2_	320.51	-	-
cis-1-Chloro-9-octadecene	32.836	1.29	C_18_H_35_Cl	286.9	-	-
Hexadecanoic acid, 2-hydroxy-1-(hydroxymethyl)ethyl ester	33.125	1.96	C_19_H_38_O_4_	330.50	Antimicrobial, antioxidant, pesticide, hemolytic	[42]
Docosyl ethyl carbonate	33.307	2.37	C_25_H_50_O_3_	398.66	-	
Phthalic acid, di(2-propylpentyl) ester	33.450	1.50	C_24_H_38_O_4_	390.55	Anticancer	[43]
Tetrapentacontane, 1,54-dibromo-	34.588	1.20	C_54_H_108_Br_2_	917.2	Can treat chronic illnesses	[44]
3-Ethyl-3-methylnonadecane	36.797	1.24	C_22_H_46_	310.6	-	-
Stigmast-5-en-3-ol, oleate	37.965	1.30	C_47_H_82_O_2_	679.2	-	-

**Table 4:** Zone of inhibition (including 6 mm well diameter and excluding 6 mm well diameter) of *Streptomyces* sp. KS20 EtOAc-Ex against bacterial pathogens.

	Zone of Inhibition (mm)			
	Including 6 mm Well Diameter		Excluding 6 mm Well Diameter	
Pathogens	Standard	EtOAc-Ex	Standard	EtOAc-Ex
*S. aureus*	30 ± 0.93	23 ± 0.83	24 ± 0.93	17 ± 0.83
*B. cereus*	33 ± 1.24	21 ± 1.46	27 ± 1.24	15 ± 1.46
*B. subtilis*	34 ± 0.98	26 ± 1.05	28 ± 0.98	20 ± 1.05
*E. faecalis*	32 ± 0.69	27 ± 1.01	26 ± 0.69	21 ± 1.01
*E. coli*	33 ± 0.36	36 ± 0.83	27 ± 0.36	30 ± 0.83
*P. aeruginosa*	28 ± 1.25	23 ± 1.16	22 ± 1.25	17 ± 1.16
*S. flexneri*	33 ± 0.97	24 ± 1.16	27 ± 0.97	18 ± 1.16
*K. pneumoniae*	33 ± 0.39	19 ± 1.14	27 ± 0.39	13 ± 1.14

Note: The results are ± SD of three independent replicates.

**Table 5:** MIC and MBC of EtOAc-Ex of *Streptomyces* sp. KS20 against bacterial pathogens.

Broth Dilution Assay		
Pathogens	MIC (µg/mL)	MBC (µg/mL)
*S. aureus*	3.12	6.25
*B. cereus*	6.25	12.5
*B. subtilis*	3.12	3.12
*E. faecalis*	3.12	6.25
*E. coli*	12.5	25
*P. aeruginosa*	12.5	25
*S. flexneri*	3.12	6.25
*K. pneumoniae*	12.5	25

## Data Availability

The data related to *Streptomyces* sp. KS20 are available at NCBI with accession number ON908964, while other data are publicly unavailable due to privacy.
